# Hyperactive ice‐binding proteins stabilize cell membranes and improve resistance to dehydration stress in *Caenorhabditis elegans*

**DOI:** 10.1002/2211-5463.70274

**Published:** 2026-05-21

**Authors:** Daiki Shimose, Kotaro Ozaki, Ryohei Kuriyama, Yuka Ikemoto, Kazuhiro Mio, Yuji C. Sasaki, Tatsuya Arai, Sakae Tsuda, Yoichi Shinkai, Masahiro Kuramochi

**Affiliations:** ^1^ Graduate School of Science and Engineering Ibaraki University Japan; ^2^ Spectroscopy and Imaging Division Japan Synchrotron Radiation Research Institute Sayo‐gun Japan; ^3^ AIST‐UTokyo Advanced Operando‐Measurement Technology Open Innovation Laboratory (OPERANDO‐OIL) National Institute of Advanced Industrial Science and Technology (AIST) Kashiwa Japan; ^4^ Graduate School of Frontier Sciences The University of Tokyo Kashiwa Japan; ^5^ Graduate School of Life Science Hokkaido University Sapporo Japan; ^6^ Cellular and Molecular Biotechnology Research Institute, National Institute of Advanced Industrial Science and Technology (AIST) Tsukuba Japan

**Keywords:** *C. elegans*, cell membrane, dehydration stress, ice‐binding protein

## Abstract

Ice‐binding proteins (IBPs) are known to modulate ice growth and promote freezing tolerance and have recently attracted attention due to their cell‐protective functions under freezing or low‐temperature conditions. In this study, we demonstrate that IBP expression improves resistance to dehydration stress in *Caenorhabditis elegans*. After 30 min of drying, transgenic worms expressing a high‐activity fungal IBP (TisIBP8) showed a modest but significant increase in survival compared with wild‐type worms. Furthermore, imaging revealed reduced muscle‐cell damage in the TisIBP8‐expressing worms while synchrotron radiation infrared microspectroscopy showed membrane structural changes during dehydration were attenuated by TisIBP8 expression. These findings suggest that IBPs exert protective effects by stabilizing cell membranes independent of ice binding, therefore broadening the potential applications of IBPs for biological preservation under nonfreezing stress conditions.

## Graphical abstract

TisIBP8, a fungal‐derived hyperactive ice‐binding protein, helps *Caenorhabditis elegans* survive dehydration. It localizes near cell membranes, reduces cell damage, and helps maintain membrane structure during drying. These results suggest that ice‐binding proteins can protect cells from dehydration stress as well as freezing stress.

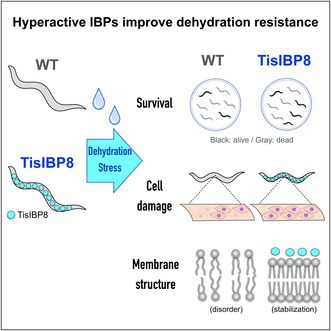

**Abbreviations**
IBP: Ice‐binding protein
IR: Infrared
NGM: Nematode growth medium
RH: Relative humidity

Ice‐binding proteins (IBPs) are proteins that bind to ice crystal surfaces and thereby modulate ice growth, morphology, and recrystallization, often contributing to freeze tolerance and cryoprotection [1, 2]. They have been identified in a wide range of taxa, including fishes, insects, plants, and fungi. The binding planes on the ice crystals and the resulting crystal morphologies differ depending on the type of IBP. For example, fish‐derived IBPs often reshape ice crystals into needle‐like structures [3, 4], whereas highly active IBPs derived from insects and fungi more strongly inhibit ice growth and generate rounded hexagonal crystals [2, 5]. The ability of IBPs to suppress ice crystal growth can be quantified as thermal hysteresis (TH). TH is defined as the difference between the melting point and the nonequilibrium freezing point of a solution containing an IBP, and it is correlated with the physiological protective effects of IBPs [5, 6, 7].

In recent years, alongside structural and biophysical analyses of IBPs and their interactions with ice, increasing attention has been given to evaluating their effects *in vivo*. In the model organism *Caenorhabditis elegans*, which naturally lacks IBPs, the introduction of genes encoding IBPs, such as the fish‐derived NfeIBP and fungus‐derived *Typhula ishikariensis* TisIBP8 and AnpIBP has been shown to improve survival following cryopreservation, indicating that IBP expression can confer freezing tolerance [8, 9]. Among these IBPs, TisIBP8, as shown in Fig. S1 (refs [10, 11]), is particularly notable because of its exceptionally high ice‐binding activity [12] and striking protective effects in worms. For example, at −5 °C, the survival rate of TisIBP8‐expressing worms was nearly 10 times that of wild‐type (WT) worms. Furthermore, X‐ray nanodynamic observation has been used to directly visualize IBPs binding to ice crystals and suppressing their growth, providing compelling evidence of their functional activity in living systems [13].

Interestingly, the protective effects of IBPs are not limited to freezing. In cultured cells supplemented with fish‐derived IBPs, improved survival has been observed at low temperatures, such as 4 °C, an effect thought to result from the specific adsorption of IBPs to cell membranes [14, 15, 16, 17, 18]. Similar improvements in cold tolerance have also been observed in *C. elegans* at 2 °C and 4 °C [8]. Moreover, IBPs with high TH activity often confer stronger protection, suggesting a potential mechanism beyond simple ice binding, namely, the stabilization of cellular membranes [14].

These findings support the working hypothesis that the principal function of IBPs extends beyond ice binding to include membrane stabilization. If IBPs can stabilize membranes, their protective scope may encompass not only nonfreezing low‐temperature stress but also other stresses in which membrane damage predominates, most notably dehydration stress. The classical mechanisms of desiccation tolerance include water replacement and vitrification by trehalose, as well as the actions of LEA (late embryogenesis abundant) proteins. More recently, the involvement of other intrinsically disordered protective protein systems, such as TDPs (tardigrade‐specific intrinsically disordered proteins) and hydrophilins, has also attracted attention. Trehalose reduces structural fluctuations by forming sugar glass with a high glass transition temperature (*T*_g_). Extensive hydrogen bonding (i.e., water replacement) preserves the lipid bilayer spacing and protein hydration shells, thereby arresting deleterious molecular motion. As a result, protein denaturation and aggregation, as well as excessive membrane packing and leakage, are suppressed [19, 20, 21, 22, 23, 24, 25, 26, 27]. In contrast, intrinsically disordered protective proteins, including LEA proteins, are generally highly hydrophilic and largely disordered in aqueous solution [28, 29], and are thought to contribute to desiccation tolerance through multiple mechanisms under drying conditions, including stabilization of proteins and membranes, suppression of denaturation and aggregation, and possible involvement in glass‐like states, phase separation, and protective condensate formation [29, 30, 31, 32, 33, 34, 35]. In addition, TDPs have been reported to protect cells during drying through vitrification, whereas hydrophilins have been shown to have broad protein‐stabilizing functions [31, 36].

Robust survival after severe desiccation in *C. elegans* has been demonstrated primarily in dauer larvae, a specialized stage with high stress resistance. This desiccation tolerance has been reported to depend on preconditioning, with trehalose playing an important role [37, 38, 39]. More recently, LEA‐1 has also been shown to contribute to tolerance against mild desiccation and osmotic stress in dauer/dauer‐like animals [40]. In contrast, nondauer animals are generally far more vulnerable to desiccation. We therefore hypothesized that IBPs, through properties such as TH activity and membrane‐binding affinity, may stabilize membrane structure and function under dehydration stress and thereby partially alleviate dehydration‐associated damage. The aim of this study was not to reproduce or validate the classical anhydrobiosis observed in dauer larvae, but rather to examine whether expression of ice‐binding proteins can partially mitigate dehydration‐associated damage in nondauer *C. elegans*. To test this hypothesis, we examined the physiological role of IBPs under dehydration stress in transgenic *C. elegans* worms expressing individual IBPs. We assessed survival after dehydration, quantified muscle‐cell damage using fluorescence imaging, and investigated membrane structural changes using synchrotron radiation infrared microspectroscopy. These experiments revealed that TisIBP8, a high‐activity fungal IBP, substantially reduced lethality under dehydration stress and helped preserve cellular structural integrity.

## Materials and methods

### Plasmid construction and generation of transgenic lines

To express the TisIBP8–wrmScarlet fusion protein in body‐wall muscle cells, we modified an existing construct, *myo‐3p::TisIBP8* (based on the pPD95.79 vector, kindly provided by Andrew Fire), where wrmScarlet is a red fluorescent protein. First, the wrmScarlet gene with its stop codon removed was inserted between the KpnI and EcoRI sites in the vector. Next, the TisIBP8 gene, flanked by EcoRI sites at both the 5′ and 3′ ends, was inserted into the EcoRI site. All inserted sequences were verified using DNA sequencing to ensure accuracy. The final plasmid construct was introduced into *C. elegans* using the microinjection method described by Mello *et al*., [41] and a stable transgenic line was established.

The *C. elegans* strains used in this study included the WT N2 (Bristol) strain, previously established transgenic lines developed in our laboratory [8], and a newly generated transgenic strain. All *C. elegans* strains used in this study are listed in Table S1 (ref. [8, 42, 43, 44, 45, 46, 47]).

In this study, a body‐wall muscle‐specific IBP‐expressing strain was used. In our previous study, comparison of strains expressing IBPs in body‐wall muscle, neurons, and intestine showed that the body‐wall muscle‐expressing strain had the highest tolerance to cold and freezing stress [8]. In addition, because the locomotor phenotype used for viability scoring was easier to evaluate in this strain and because it was also suitable for cell‐level analyses by fluorescence imaging and infrared spectroscopy, the body‐wall muscle‐specific expression system was adopted in this study.

### Dehydration stress assay in *C. elegans*

Worms were cultured from the egg stage to the larval L4 stage (L4) or young adult stage at 22 °C on nematode growth medium (NGM) plates seeded with OP50. For the assay, several hundred adult worms were washed off the NGM plates with 1 mL of M9 buffer, and the worm suspension was collected. The suspension was transferred to a 1.5 mL microcentrifuge tube and centrifuged at 3000× ***g*** for 3 min at 4 °C to pellet the worms. After the supernatant was removed, 5 μL of the worm pellet was spotted onto a 6‐cm Petri dish (without agar). The dish, with its lid removed, was immediately placed in a vacuum desiccator (RVD‐250; AS ONE Corporation; Osaka, Japan) containing silica gel (FUJIFILM Wako Pure Chemical Corporation; Osaka, Japan) as a drying agent. The treatment was performed at room temperature, and the relative humidity inside the desiccator was adjusted to approximately 20–30% by adding silica gel as needed. Dehydration treatment was performed for 10, 20, 30, or 40 min. After each drying period, the dish was removed from the desiccator and rehydrated by adding 1 mL of M9 buffer. Worm viability was assessed by the presence of spontaneous head swings (head movements) following rehydration, and worms exhibiting such movements were considered alive.

### Thrashing assay

The thrashing assay was performed by placing a single L4 or adult worm, previously dried for 30 min, into M9 buffer and manually counting body bends (thrashes) for 1 min.

### Cell count assay

As *C. elegans* has a transparent body, individual cells and tissues can be easily visualized under a microscope. To evaluate the cell‐protective effects of IBPs under dehydration stress, we generated transgenic worms expressing nucleus‐localized red fluorescent protein (NLS::wrmScarlet) in body‐wall muscle cells and quantified the number of surviving cells. wrmScarlet was localized to the nucleus via a nuclear localization signal (NLS) and was visualized as discrete fluorescent puncta, enabling the identification and manual counting of individual muscle cells *in vivo* (Fig. S2). Mammalian skeletal muscle fibers are multinucleated, whereas the body‐wall muscle cells of *C. elegans* are mononucleate. Consequently, counting muscle‐cell nuclei in *C. elegans* provides a direct quantification of cell number. For observation, a single worm was placed on a 2.0% agarose pad on a glass slide with 5 μL of M9 buffer containing 50 mm sodium azide. The slide was immediately placed in a desiccator for 30 or 60 min to induce dehydration. After drying, a coverslip was applied, and fluorescence imaging was performed. Images were acquired using an inverted fluorescence microscope (IX71; Olympus, Tokyo, Japan) equipped with a 40× objective lens, and the fluorescent nuclei were manually counted. The numbers of nuclei before and after dehydration treatment were compared, and the ratio was calculated as nuclear retention (%) = 100 × (*N*_post_/*N*_pre_), where *N*_pre_ and *N*_post_ represent the number of nuclei before and after dehydration treatment, respectively.

### Water loss assay

Synchronized young adult worms (100 individuals per replicate) were collected individually using a platinum wire picker and washed three times with M9 buffer to remove bacteria. Hydrophilic polycarbonate Isopore membranes (pore size 8.0 μm, diameter 25 mm; Merck, Darmstadt, Germany) were placed in aluminum dishes, and the worms were transferred onto the membranes. Excess buffer was removed by blotting, and the initial wet mass was recorded using a microbalance. The samples were then placed in a desiccation chamber containing silica gel and kept at room temperature (relative humidity < 20%) for 30 min, after which the final dry mass was measured. The membrane mass was predetermined and subtracted from all the measurements. Water loss was calculated as the difference between the wet and dry masses per 100 worms, with buffer‐only controls processed in parallel. A total of 13–15 biological replicates were used.

### Confocal imaging of intracellular IBP localization

Adult worms were anesthetized in an M9 solution containing 20 mm sodium azide and immobilized on a 1.5% agarose pad. Images were acquired using an inverted confocal microscope (A1; Nikon, Tokyo, Japan) equipped with a 60× objective and were processed using nis‐elements c/nis‐elements c‐er and fiji (an imagej distribution). Cross‐sectional images of the muscle cells were generated in fiji, and intensity values were obtained from line scans to calculate the ratio of the central region to the peripheral region.

### Synchrotron radiation infrared microspectroscopy

To evaluate the structural properties of cell membranes, synchrotron radiation infrared (IR) microspectroscopy was performed. The measurements were conducted at beamline BL43IR at SPring‐8, a large‐scale synchrotron radiation facility in Hyogo, Japan. This beamline enables the focusing of infrared light to the micrometer scale, allowing structural analysis at the single‐cell level in *C. elegans*. Samples were prepared by placing approximately 3 μL of 10% D_2_O/H_2_O solution at the center of a 10 mm diameter, 1 mm thick barium fluoride (BaF_2_) substrate, into which several adult worms were transferred. D_2_O was added to help distinguish the spectra of intracellular origin from those of extracellular origin. A spacer with an ~7 mm diameter hole was then carefully placed on top of the BaF_2_ substrate to prevent the worms from being crushed between the BaF_2_ plates and killed. Another BaF_2_ substrate was placed over the spacer to seal the sample containing the D_2_O/H_2_O solution. Although the thickness of a *C. elegans* worm is approximately 30–50 μm, a 7‐μm‐thick spacer was used to minimize contributions from the surrounding solution and ensure the acquisition of spectra within the worm body. The assembled sample was mounted into a liquid sample cell (DLC‐M25; Harrick Scientific, NY, USA) and measured in transmission mode using a high‐spatial‐resolution IR microscope (Bruker Hyperion 2000; Bruker, MA, USA). A 15× objective was used for observations, and all measurements were conducted at room temperature. BaF_2_ was used as the window material on both sides of the cell, and an mercury cadmium telluride (MCT) detector was employed for spectral detection. The initial spectra were recorded under controlled (predrying) conditions. The sample cells were then removed from the microscope stage and placed in a desiccator for drying. Because of the ambient humidity in the facility, it was difficult to maintain the relative humidity below 40%; therefore, a fixed drying duration of 40 min was used for the evaluation. After drying, the sample cell was returned to the microscope stage, and the spectra were acquired again.

### Statistical analyses

All the statistical analyses were performed using R version 3.3.1, and statistical significance was evaluated using Student's *t*‐test.

## Results and discussion

### The hyperactive ice‐binding protein TisIBP8 improved resistance to dehydration stress in *C. elegans*

**Fig. 1:**
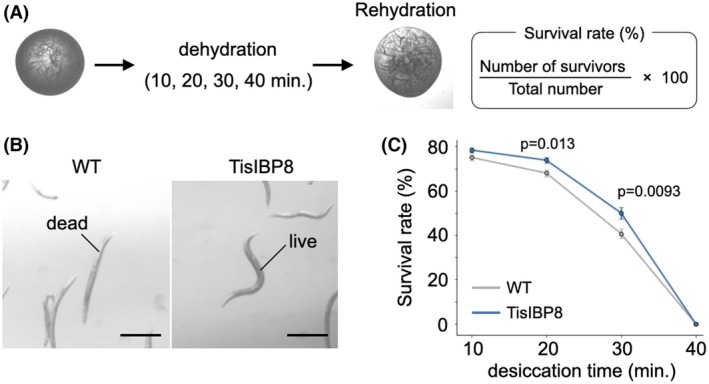
TisIBP8 improves the survival of *C. elegans* under dehydration stress. (A) Schematic diagram of the dehydration and rehydration assay. Worms (L4 larvae and adults) were subjected to air‐drying in a droplet and were then rehydrated. (B) Representative images of wild‐type (WT) and TisIBP8‐expressing worms after dehydration treatment. The scale bar represents 0.5 mm. (C) Survival rates of WT and TisIBP8 worms after dehydration treatment for different durations (10, 20, 30, and 40 min). Data are presented as the mean ± SEM values. The number of worms scored per replicate ranged from 20 to 163. The numbers of biological replicates were *n* = 20 for both groups at 10 and 20 min, *n* = 30 for WT and *n* = 31 for TisIBP8 at 30 min, and *n* = 7 for both groups at 40 min. Student's *t*‐test.

Dehydration, low temperatures, extreme osmotic or thermal conditions, oxidative stress, and mechanical strain can perturb membrane phase behavior and promote ion leakage [48, 49]. It is well known that stabilizing the physical state and permeability of the membrane endows broad stress tolerance. Introduction of IBP genes improves freezing and cold tolerance in various organisms [8, 50]. More recently, IBPs have been reported to be effective also under cold but nonfreezing conditions, possibly because they protect cells through adsorption to the plasma membrane [17]. If such membrane‐associated protection mechanisms exist, IBPs could be effective against a broader range of environmental stresses beyond freezing and cold. To explore this possibility, we investigated the effect of IBP expression on resistance to dehydration stress in *C. elegans*. WT worms and IBP‐expressing transgenic worms were placed in droplets and allowed to dry in a desiccator (relative humidity, 20%), with drying periods ranging from 10 to 40 min in 10‐min increments. After each drying period, water was added to rehydrate the samples, and survival was assessed by observing whether the worms had resumed movement (Fig. 1A,B). The survival rates of the WT worms after 10, 20, and 30 min of drying were 75 ± 1%, 68 ± 2%, and 41 ± 2%, respectively. In contrast, worms expressing the high‐activity variant TisIBP8 had higher survival rates, namely 78 ± 1%, 74 ± 1%, and 50 ± 3% after 10, 20, and 30 min, respectively. Notably, at 20 and 30 min, the survival rates of TisIBP8‐expressing worms were significantly higher than those of WT worms (Fig. 1C and Table S2). At 40 min, no surviving individuals were observed in either of the groups. These results suggest that TisIBP8 mitigates dehydration‐induced damage and offers a modest survival benefit but does not confer resistance sufficient to prevent death under severe drying conditions. In addition, when TisIBP8 was tested at a lower concentration, no significant improvement in survival was observed compared with WT (Fig. S3). This result suggests that the protective effect of TisIBP8 may depend on its effective expression level or concentration.

Because TisIBP8 showed a protective effect under dehydration stress, we next examined whether other IBPs, such as NfeIBPs (fish‐derived) and AnpIBPs (fungus‐derived), also showed similar protective effects. NfeIBPs were classified as low‐activity proteins and AnpIBP as a medium‐activity protein, with AnpIBP T156Y being a low‐activity variant. These assays were conducted separately from the initial experiments using TisIBP8, and because of experimental constraints, the relative humidity (RH) was 33%. Under these conditions, the survival rate of WT worms was 47 ± 2%, which was higher than that at 20% RH. In contrast, the survival rates of the strains expressing NfeIBP6, NfeIBP8, AnpIBP, and the AnpIBP T156Y variant were 49 ± 3%, 47 ± 2%, 50 ± 2%, and 50 ± 2%, respectively, with no significant differences from that of the WT strain (Fig. S4). These results suggest that low‐ and medium‐activity IBPs did not provide clear protection under these conditions, whereas a modest improvement in dehydration resistance was observed for the high‐activity TisIBP8.

### IBPs may suppress muscle‐cell damage caused by dehydration stress

**Fig. 2:**
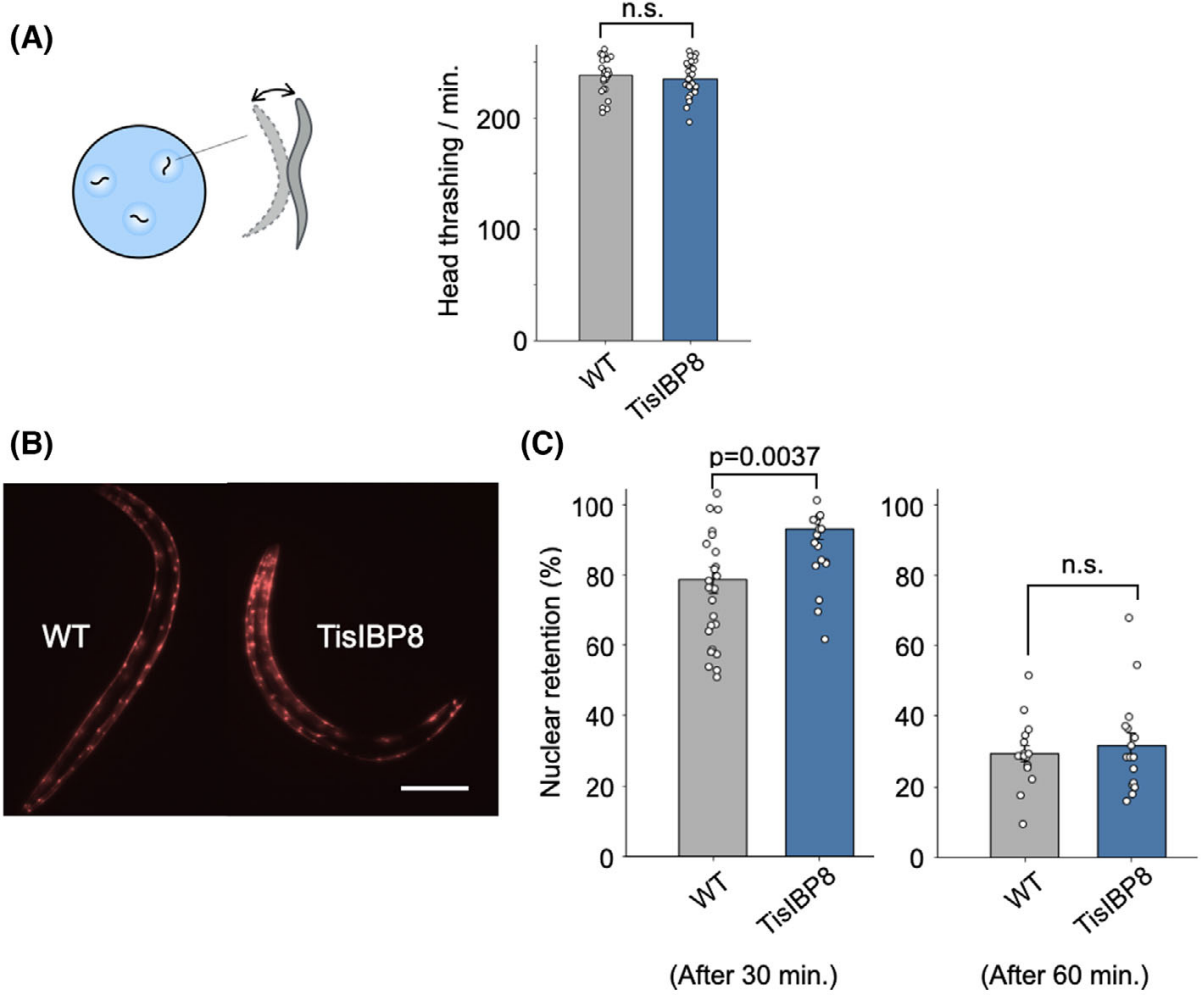
TisIBP8 expression protects muscle cells from dehydration‐induced damage in *C. elegans*. (A) A head thrashing assay was performed to evaluate muscle function 30 min after dehydration treatment. Data are presented as the mean ± SEM values. Student's *t*‐test. n.s: not significant. (B) Fluorescence imaging of body‐wall muscle cells expressing nucleus‐localized red fluorescent protein. Scale bar is 0.2 mm. (C) The number of muscle‐cell nuclei was determined before and after dehydration treatment, and the ratio was calculated. Data are presented as the mean ± SEM values. Biological replicate numbers were *n* = 28 (WT) and *n* = 24 (TisIBP8) at 30 min, and *n* = 16 for both groups at 1 h. Welch's *t*‐test.

The increased survival rates observed under dehydration stress suggested that cellular damage may also be mitigated. In this study, because IBP was specifically expressed in body‐wall muscle cells, it is likely that the protective effect of IBP contributes to the preservation of muscle‐cell integrity. To test this hypothesis, we first evaluated thrashing movement, a commonly used indicator of muscle function. The numbers of body bends per minute for WT and TisIBP8‐expressing worms were 238.2 ± 2.7 and 234.9 ± 2.8, respectively, with no significant differences observed (Fig. 2A). These results suggest that overall motor function was largely preserved after dehydration treatment. However, while thrashing movement reflects global muscle activity, it is not a sufficient parameter for assessing damage or structural changes at the cellular level in individual cells.

To evaluate the morphological impact of dehydration stress on muscle cells, we used transgenic *C. elegans* worms expressing the red fluorescent protein wrmScarlet in muscle nuclei, which is visualized as distinct fluorescent puncta suitable for accurate quantification (Fig. 2B). In WT worms, the proportion of detectable nuclear signals decreased to ~78.4 ± 3.5% of the initial value after 30 min of drying. In contrast, worms expressing TisIBP8 retained ~92.9 ± 3.0% of their detectable nuclear signals, and the difference between WT and TisIBP8‐expressing worms was statistically significant (Fig. 2C and Table S3). These results suggest that TisIBP8 mitigates drying‐induced structural damage in muscle cells. After 60 min of drying, nuclear retention was 29.4 ± 2.4% in WT worms and 31.7 ± 3.4% in TisIBP8‐expressing worms, with no significant difference between the two groups (Fig. 2C and Table S3). The proportion of detectable nuclei was markedly reduced, consistent with severe structural damage caused by drying and loss of detectable fluorescent signal.

### IBP stabilizes membrane structure and mitigates dehydration‐induced damage

**Fig. 3:**
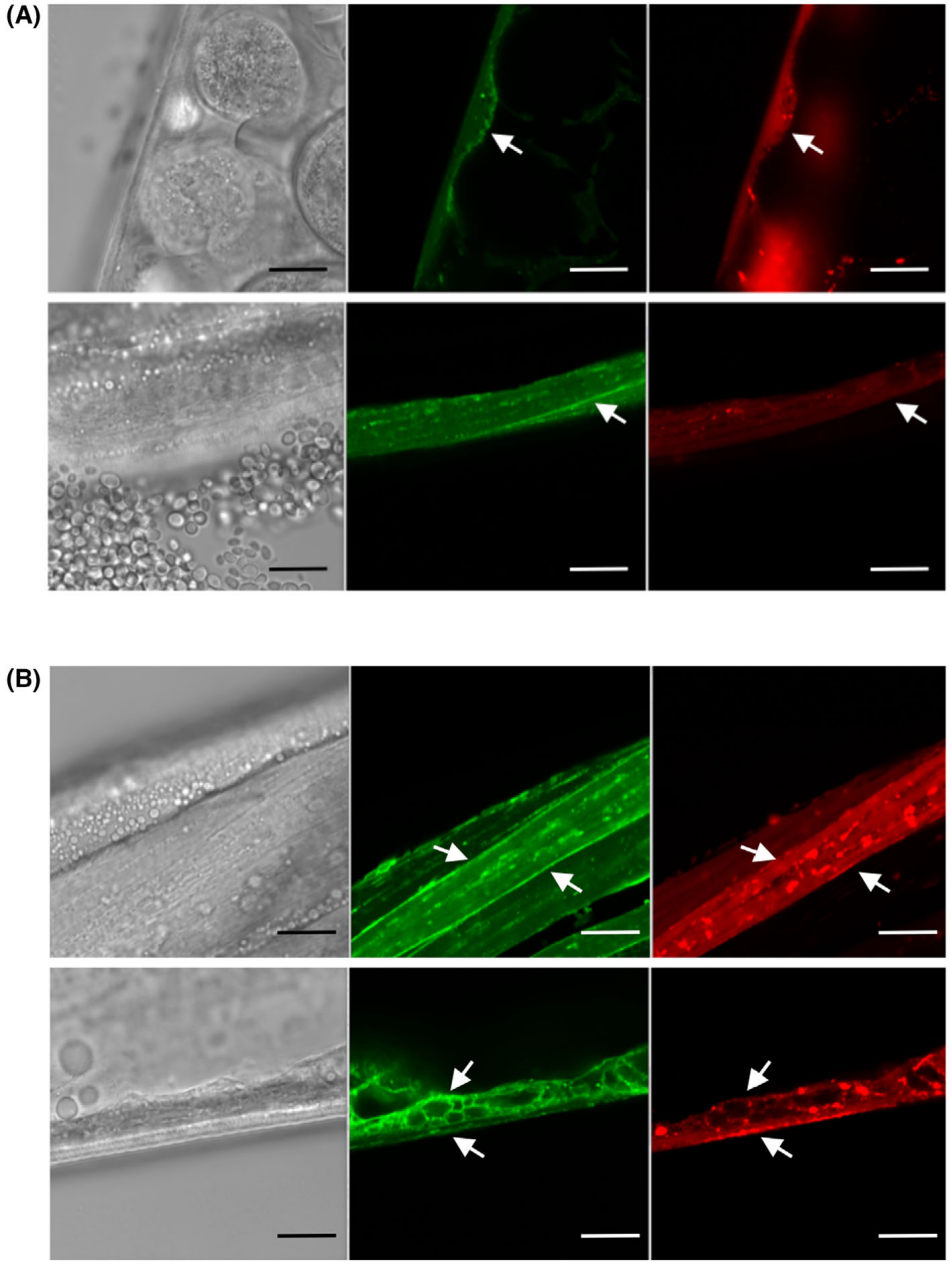
Comparison of subcellular localization between wrmScarlet alone and IBP–wrmScarlet in body‐wall muscle cells of *C. elegans*. (A) Representative images of worms expressing wrmScarlet alone in body‐wall muscle cells. Bright‐field, myristoylated GFP fluorescence (green), and wrmScarlet fluorescence (red) images are shown. (B) Representative images of worms expressing the IBP–wrmScarlet fusion protein in body‐wall muscle cells. Bright‐field, myristoylated GFP fluorescence (green), and wrmScarlet fluorescence (red) images are shown. A membrane‐proximal fluorescence pattern was observed for the IBP–wrmScarlet fusion protein. Arrows indicate the membrane region. Scar bar is 10 μm.

The above results strongly suggest that IBPs suppress cellular damage during dehydration stress. However, the precise mechanism underlying this protective effect remains unclear. Previous researchers have proposed that IBPs perform their protective function by specifically adsorbing onto cellular membranes [17, 47]. To evaluate this possibility, we expressed a fusion of IBP and the red fluorescent protein wrmScarlet (TisIBP8–wrmScarlet) specifically in body‐wall muscle cells and examined its intracellular distribution by confocal microscopy. To define the membrane region more clearly, we generated worms co‐expressing myristoylated GFP and examined the fluorescence together with the membrane‐marker signal. Fluorescence was broadly observed throughout the cytoplasm, but additional imaging using a membrane marker suggested that TisIBP8–wrmScarlet was biased toward the membrane‐proximal region, whereas such a pattern was not observed for wrmScarlet alone (Fig. 3). For a more quantitative assessment, we performed image analysis on body‐wall muscle images from worms expressing wrmScarlet alone (WT) and from worms expressing TisIBP8–wrmScarlet (TisIBP8) (Fig. S5A,B). The pseudo‐cross‐sectional intensity profiles generated by summing the single‐cell images differed between the two groups; the relative intensity at the section edge (membrane‐proximal region) tended to be greater in TisIBP8‐expressing worms (Fig. S5C). Consistent with this finding, the ratio of the membrane‐proximal to the central intensity (edge/center) was significantly higher in TisIBP8‐expressing worms than in WT worms, suggesting the possibility that the IBP is associated with the membrane‐proximal region (Fig. S5D).

Given the structural features of IBPs and their high affinity for water molecules, it is also possible that IBPs exert protective effects by retaining water within cells. To evaluate this possibility, we evaluated the water loss rate of WT and TisIBP8‐expressing worms before and after dehydration treatment. First, the water content in the drying assay plate decreased over time, and the relative water content fell below 10% at both 30 and 40 min, indicating that it had reached an almost saturated low level (Fig. S6A). The samples were considered to be nearly completely dry by 30 min. If the IBPs retain intracellular water, we expected less weight loss after drying. However, the water loss rates were similar between WT and TisIBP8‐expressing worms (WT: 91.1 ± 0.9%; TisIBP8: 91.5 ± 0.8%), and no statistically significant difference was observed between the two groups (Fig. S6B). These results suggest that water retention is unlikely to be the primary mechanism through which IBPs confer protection under dehydration stress.

**Fig. 4:**
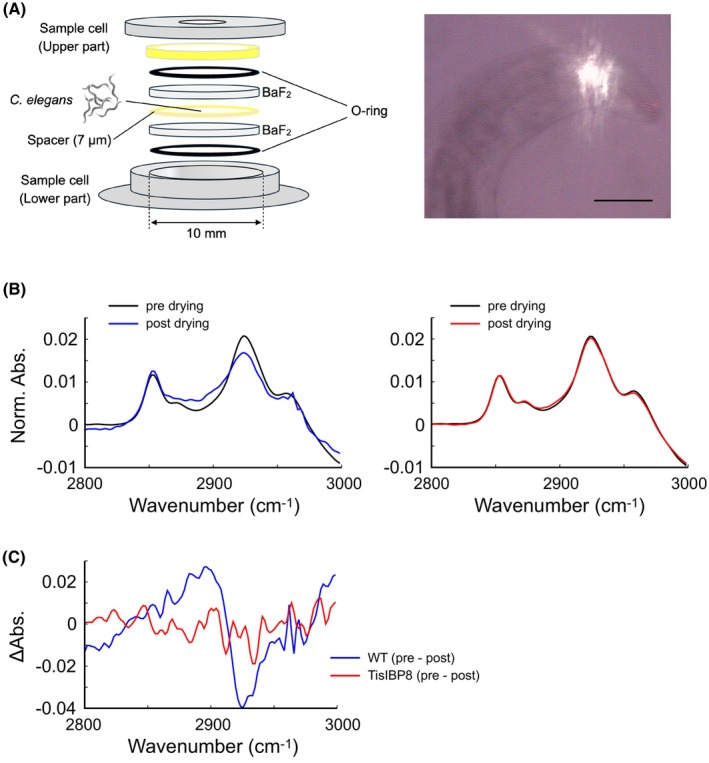
Infrared spectroscopic analysis of membrane structure before and after dehydration treatment. (A) Schematic of the BaF_2_ sandwich cell used for synchrotron radiation IR microspectroscopy of *C. elegans* worms (left) and a representative micrograph of a worm positioned for measurement (right). Scale bar is 0.2 mm. (B) Mean normalized infrared (IR) absorption spectra in the C—H stretching region (2800–3000 cm^−1^) acquired from wild‐type (WT, left; *n* = 12) and TisIBP8‐expressing (right; *n* = 11) worms before and after 40 min of dehydration treatment. The bands at ~ 2920 and ~ 2850 cm^−1^ correspond to the antisymmetric and symmetric CH_2_ stretching vibrations, respectively, of lipid acyl chains. (C) Difference spectra (postdrying–predrying) for WT (blue) and TisIBP8‐expressing (red) worms.

Next, using infrared (IR) spectroscopy, we assessed whether IBPs influence membrane structural integrity. To acquire high‐resolution spectra from muscle‐cell membranes, we utilized synchrotron radiation IR microspectroscopy (beamline BL43IR at SPring‐8), which enables IR light to be focused on the micrometer scale (Fig. 4A). Spectra were obtained from WT and TisIBP8‐expressing worms before and after 40 min of dehydration stress at 40% relative humidity, focusing on the 2800–3000 cm^−1^ region corresponding to the C—H stretching vibrations of membrane lipid acyl chains. Notably, the RH condition in this experiment differs from that used in the survival assays, owing to experimental constraints that prevented a further reduction in RH. In WT worms, the intensities of the peaks around 2920 and 2850 cm^−1^ increased after drying, indicating significant disruption of the lipid order. In contrast, TisIBP8‐expressing worms displayed similar peaks but exhibited minimal changes in absorbance (Fig. 4B). Subtracting the predrying spectrum from the postdrying spectrum to generate differential spectra revealed pronounced spectral shifts in the WT worms but much smaller changes in the TisIBP8 worms (Fig. 4C). These results suggest that TisIBP8 expression partially suppresses dehydration‐induced disruption of membrane structure. This effect may contribute to improved resistance to dehydration stress and to the reduction in cellular damage.

In this study, we demonstrated that the expression of a high‐activity IBP, TisIBP8, improved resistance to dehydration stress in *C. elegans*. Compared with WT control worms, transgenic worms expressing TisIBP8 exhibited significantly higher survival rates under dehydration stress conditions and showed reduced muscle‐cell damage. Furthermore, synchrotron radiation IR microspectroscopy revealed that IBP expression mitigated the structural disruption of membrane lipids under dehydration stress. These findings suggest that IBPs confer protective effects independent of their classical ice‐binding function, likely through stabilization of the membrane structure.

This interpretation may also help explain previous findings that IBPs improve cryopreservation outcomes. In general, IBPs are well known to inhibit ice crystal growth and suppress recrystallization. During freezing, however, extracellular ice formation causes freeze concentration, which increases osmotic stress and reduces the amount of unfrozen water available to cells, thereby exposing them to severe dehydration. It has been reported that such freeze‐induced dehydration can cause substantial cellular damage [51]. From this perspective, the dehydration‐protective effect of IBPs observed in the present study, namely stabilization of lipid structure, may also contribute beneficially to cryopreservation, not only through control of ice crystals but also through mitigation of cell damage caused by dehydration during freezing. In other words, in addition to their role in ice control, IBPs may also function as protective factors that reduce dehydration stress associated with freezing. This membrane‐stabilizing function broadens the physiological significance of IBPs and highlights their potential utility in biological preservation beyond freezing and low‐temperature stress. This membrane‐stabilizing function broadens the physiological significance of IBPs and highlights their potential utility in biological preservation beyond freezing and low‐temperature stress.

*C. elegans* does not survive severe desiccation; however, under environmental stresses, such as starvation, they develop into dauer larvae and acquire resistance to desiccation [37]. During this process, intracellular trehalose levels increase markedly and have been reported to protect cellular structures, including membranes. Trehalose supports resistance to desiccation by physically stabilizing membranes and proteins via water replacement and vitrification [52, 53]. LEA proteins have been reported to contribute to maintaining protein structure, prevent aggregation, temper the progression of phase separation, and protect membranes and nucleic acids [30]. Although some LEA proteins have also been reported to interact with (adsorb to) membranes [54, 55], their principal roles in dehydration tolerance are still being actively investigated. The observation that IBPs adsorb to cell membranes during dehydration stress and suppress irreversible structural damage suggests that they may contribute to protective responses via mechanisms distinct from those of trehalose and LEA proteins. Combining IBPs with trehalose or LEA proteins may provide complementary and potentially synergistic protective effects.

It should be noted that the phenomenon shown in this study is not the same as the classical desiccation tolerance, namely anhydrobiosis, established in dauer larvae. This point should be clearly distinguished from the preconditioning‐dependent desiccation survival reported by Erkut *et al*., [37]. What we observed here was a partial reduction in dehydration stress‐related damage in nondauer *C. elegans* by IBP expression, and not the acquisition of the strong desiccation tolerance seen in dauer larvae. In fact, when evaluated under conditions similar to those used in previous studies, survival was confirmed in dauer larvae derived from the *daf‐2* mutant, whereas no clear desiccation survival was observed in WT or in TisIBP8‐expressing worms (Fig. S7; ref. [37]). Therefore, in the present study, *C. elegans* should be regarded not primarily as a system for discussing the desiccation tolerance mechanism itself, but rather as a model animal for evaluating the protective effects of IBPs *in vivo*.

Previous studies have reported that IBPs can stabilize lipid bilayers [14], suppress ion leakage [56, 57], and inhibit lipid phase transitions [58]. In the present study, we found that IBP expression may suppress membrane disruption under dehydration stress. In particular, the IR spectroscopic analysis suggested that dehydration‐induced disturbance of lipid structure was relatively small in IBP‐expressing *C. elegans*. Membrane adsorption of IBPs has also been reported, suggesting that such interactions may contribute to maintaining the structural order of lipid membranes. Among the IBPs examined in this study, a relatively clear protective effect under dehydration stress was observed only for TisIBP8, whereas no such effect was observed for the other low‐ or medium‐activity IBPs. Given that TisIBP8 shows higher TH activity than the other IBPs, it is possible that the strength of the membrane‐protective effect observed under dehydration stress is related in some way to the ice‐binding activity of the IBP. However, this idea remains hypothetical at present, and further studies will be needed to clarify the direct relationship between membrane‐binding ability and membrane stabilization.

Why might IBPs interact with lipid membranes? One possible explanation lies in their structural characteristics [47]; it has been shown that IBPs retain highly ordered, ice‐like hydration water on their molecular surfaces [43]. Interestingly, water molecules forming ice‐like hydrogen bond networks have been observed at the interface of model membranes composed of phosphatidylcholine (PC), a principal component of eukaryotic cell membranes [59, 60, 61]. These observations raise the possibility that IBPs interact with lipid membranes via a mechanism analogous to their ice‐binding activity, mediated through ice‐like hydration structures present on the membrane surface. Moreover, TisIBP8, a high‐activity fungal IBP, is known to have a stronger ice‐binding ability than fish‐derived IBPs and may also possess a more highly organized hydration shell. This property could increase its affinity not only for ice but also for lipid membranes. Such interactions with membranes may contribute to the reduction in dehydration‐induced cellular damage through stabilization of membrane lipid structure. Stabilization of membrane lipid structure by IBPs may provide a useful framework for understanding the previously reported suppression of ion leakage and phase transitions.

## Conflict of interest

The authors declare no conflict of interest.

## Author contributions

MK designed the research. MK, DS, RK, KO, KM, YCS, TA, YI, ST, and YS performed experiments. DS, YS, and MK analyzed the experimental data. MK wrote the manuscript.

## Supporting information


**Fig. S1.** Crystal structure of TisIBP8.
**Fig. S2.** Nuclear localization of wrmScarlet in body‐wall muscles.
**Fig. S3.** Survival under dehydration stress and concentration‐dependent evaluation of TisIBP8.
**Fig. S4.** Survival of *C. elegans* expressing various IBPs under dehydration stress.
**Fig. S5.** Membrane‐associated localization of TisIBP8 in body‐wall muscles of *C. elegans*.
**Fig. S6.** Evaluation of water loss in WT and TisIBP8 worms.
**Fig. S7.** Desiccation assay.
**Table S1.** Strain list.
**Table S2.** Individual survival data for WT and TisIBP8‐expressing worms after 30 min of dehydration.
**Table S3.** Individual nuclear retention data from the cell imaging analysis.

## Data Availability

The data underlying the results presented in this paper are not publicly available at this time but may be obtained from the corresponding author upon reasonable request.
