# Supplementary material for: Hyperactive ice‐binding proteins stabilize cell membranes and improve resistance to dehydration stress in Caenorhabditis elegans
Source: FEBS Open Bio. 2026 May 21:10.1002/2211-5463.70274. Online ahead of print. doi: 10.1002/2211-5463.70274 (PMC13398936; doi:10.1002/2211-5463.70274)
Supplement: Supplementary file 1 — Fig. S1. Crystal structure of TisIBP8. Fig. S2. Nuclear localization of wrmScarlet in body‐wall muscles. Fig. S3. Survival under dehydration stress and concentration‐dependent evaluation of TisIBP8. Fig. S4. Survival of C. elegans expressing various IBPs under dehydration stress. Fig. S5. Membrane‐associated localization of TisIBP8 in body‐wall muscles of C. elegans. Fig. S6. Evaluation of water loss in WT and TisIBP8 worms. Fig. S7. Desiccation assay. Table S1. Strain list. Table S2. Individual survival data for WT and TisIBP8‐expressing worms after 30 min of dehydration. Table S3. Individual nuclear retention data from the cell imaging analysis. [file FEB4-9999-0-s001.docx]

**Supplemental Information:**

**Hyperactive ice-binding proteins stabilize cell membranes and improve resistance to dehydration stress in *Caenorhabditis elegans***

Daiki Shimose^1^, Kotaro Ozaki^1^, Ryohei Kuriyama^1^, Yuka Ikemoto^2^, Kazuhiro Mio^3^, Yuji C. Sasaki^3,4^, Tatsuya Arai^5^, Sakae Tsuda^5^, Yoichi Shinkai^6^, Masahiro Kuramochi^1,4,6,*^

^1^Graduate School of Science and Engineering, Ibaraki University, Hitachi, 316-8511, Japan

^2^Spectroscopy and Imaging Division, Japan Synchrotron Radiation Research Institute, 1-1-1, Kouto, Sayo-cho, Sayo-gun, Hyogo, 679-5198, Japan

^3^AIST-UTokyo Advanced Operando-Measurement Technology Open Innovation Laboratory (OPERANDO-OIL), National Institute of Advanced Industrial Science and Technology (AIST), Kashiwa, 277-0882, Japan

^4^Graduate School of Frontier Sciences, The University of Tokyo, Kashiwa, 277-8561, Japan

^5^Graduate School of Life Science, Hokkaido University, Sapporo, 060-0810, Japan

^6^Cellular and Molecular Biotechnology Research Institute, National Institute of Advanced Industrial Science and Technology (AIST), Tsukuba, 305-8566, Japan


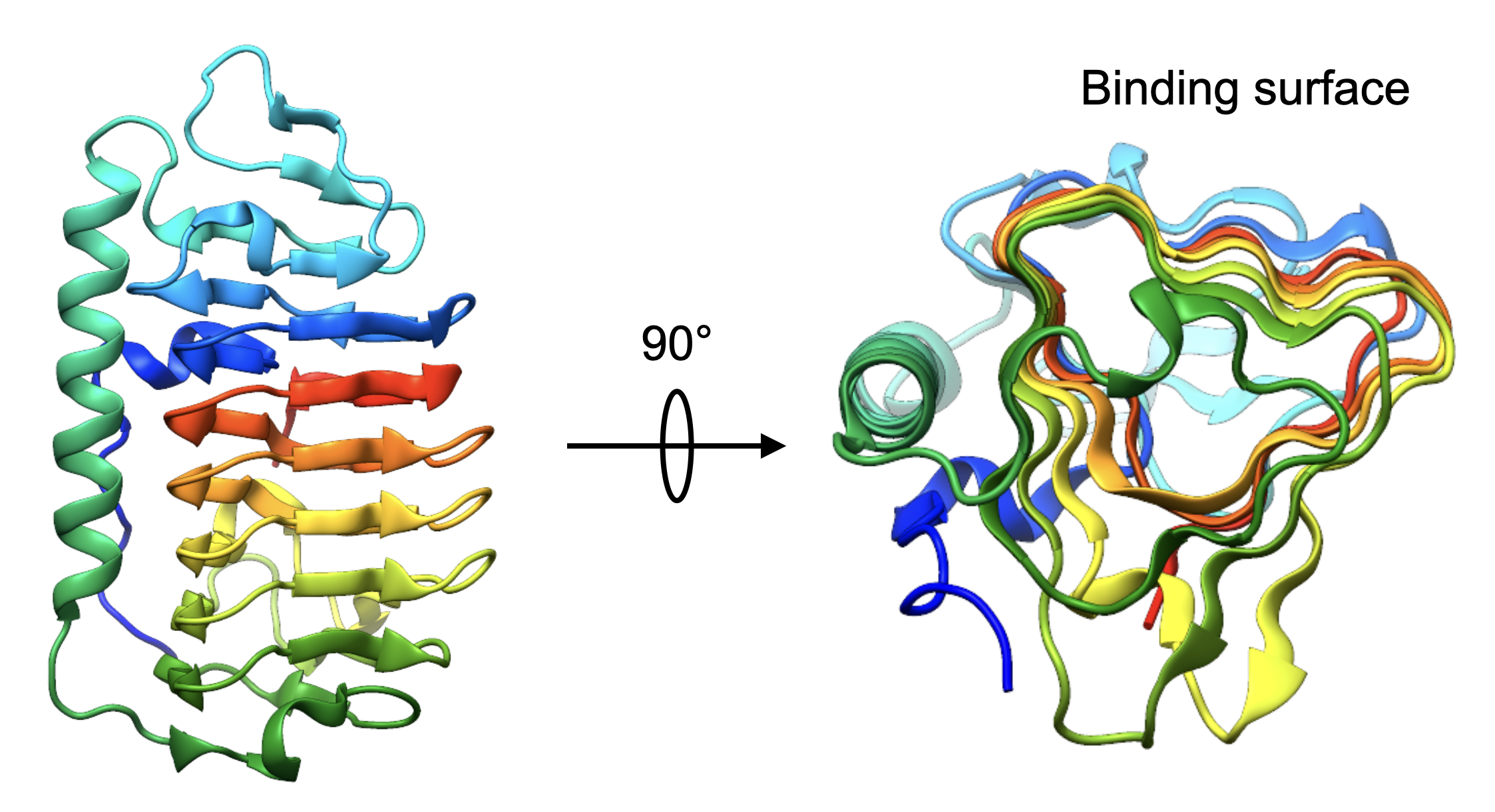


**Figure S1. Crystal structure of TisIBP8 (PDB ID: 5B5H).** The structure was obtained from the Protein Data Bank and visualized using the Mol viewer in the RCSB Protein Data Bank [10,11]. The protein is shown in ribbon representation. The color gradient represents the order of the polypeptide chain from the N-terminus to the C-terminus. The right panel shows the structure after 90° rotation, highlighting the ice-binding surface.


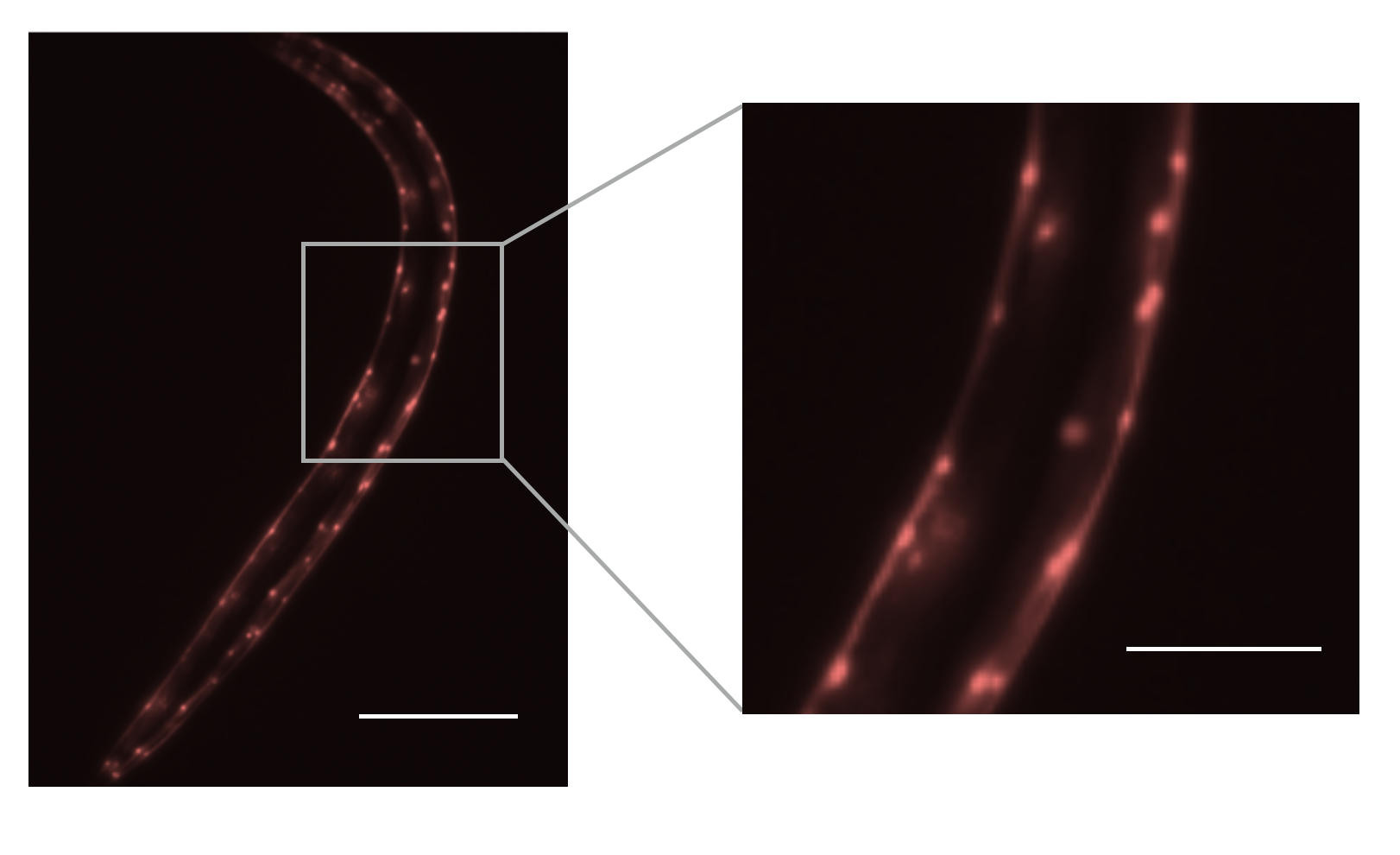


**Figure S2. Nuclear localization of wrmScarlet in body wall muscles.** Fluorescence micrograph of worms expressing the red fluorescent protein wrmScarlet fused to a nuclear localization signal (NLS) specifically in body wall muscle cells. Because fluorescence is restricted to nuclei, cells can be readily counted, enabling comparison of the numbers before and after drying. The scale bar in the original image represents 0.2 mm; in the magnified view on the right, it represents 0.1 mm.

**
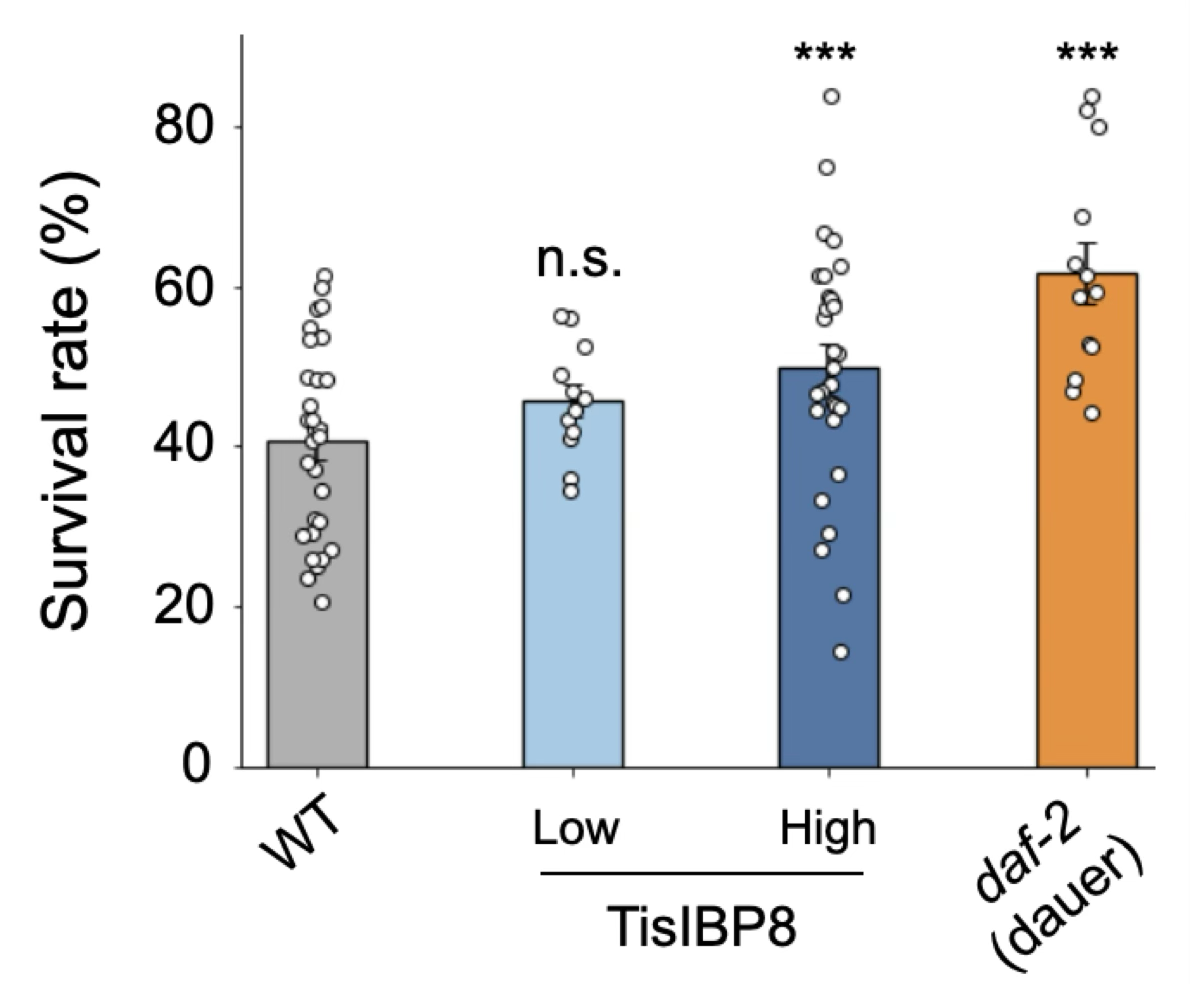
**

**Figure S3. Survival under dehydration stress and concentration-dependent evaluation of TisIBP8.** Survival rates of WT worms, worms expressing low-concentration TisIBP8, worms expressing high-concentration TisIBP8, and dauer larvae (*daf-2*) under dehydration stress. The WT and high-concentration TisIBP8 data are the same as those shown in Fig. 1C. No significant difference was observed between WT and the low-concentration TisIBP8 group. Data are presented as mean ± SEM. Each point represents one biological replicate. The numbers of biological replicates were n = 30 for WT, n = 12 for low-concentration TisIBP8, n = 31 for high-concentration TisIBP8, and n = 13 for dauer (*daf-2*). Student’s t test. ***p < 0.001


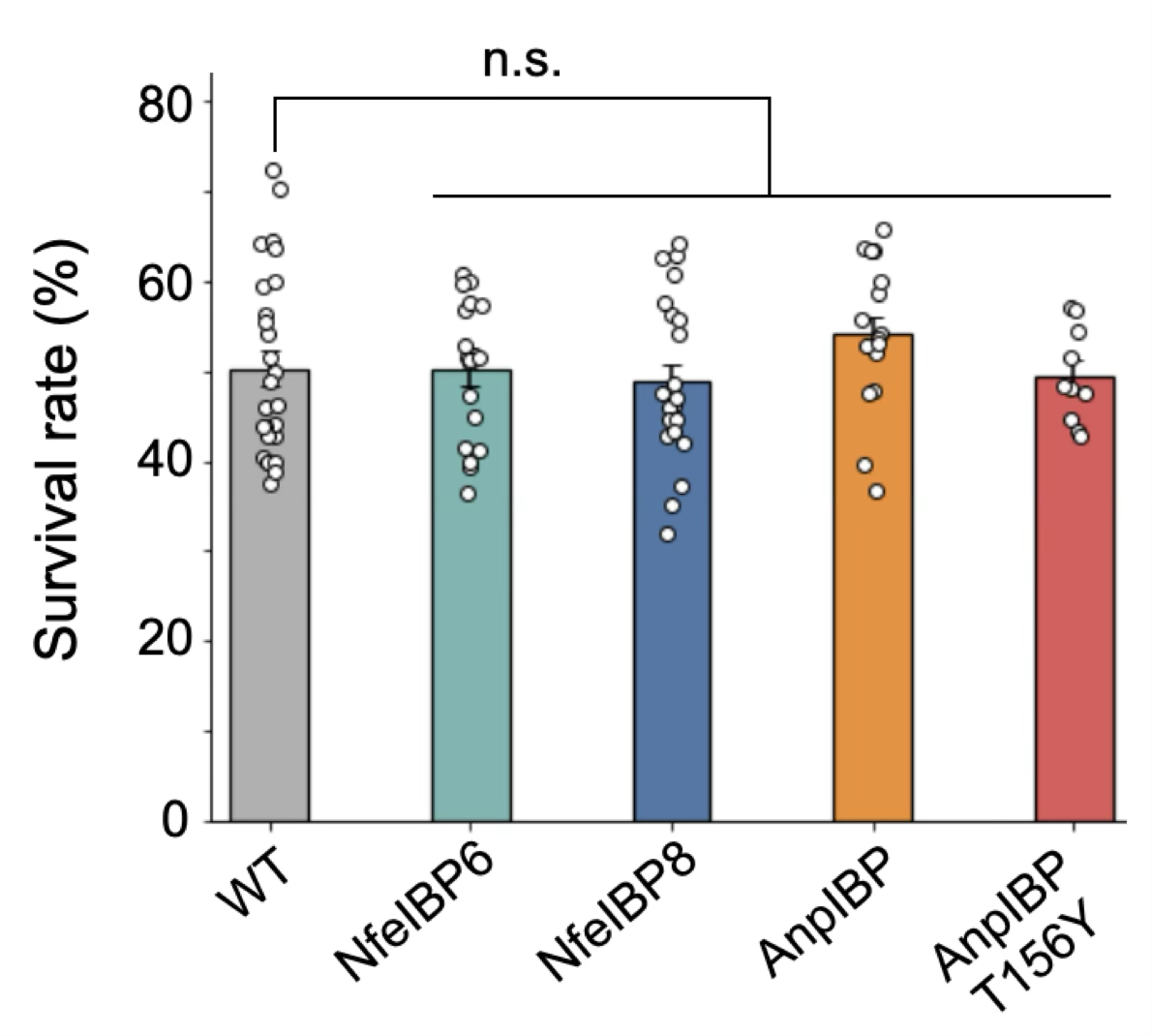


**Figure S4. Survival of *C. elegans* expressing various IBPs under dehydration stress.** Survival rates of WT worms and worms expressing different ice-binding proteins (NfeIBP6, NfeIBP8, AnpIBP, and the AnpIBP T156Y mutant) after 30 min of drying at 33% relative humidity. No clear protective effect was observed in the IBP-expressing strains compared with the WT strain under these conditions. Data are presented as mean ± SEM. Each point represents one biological replicate. At least 25 worms were scored for each group, with at least 9 biological replicates per group. Student’s t test. n.s., not significant.


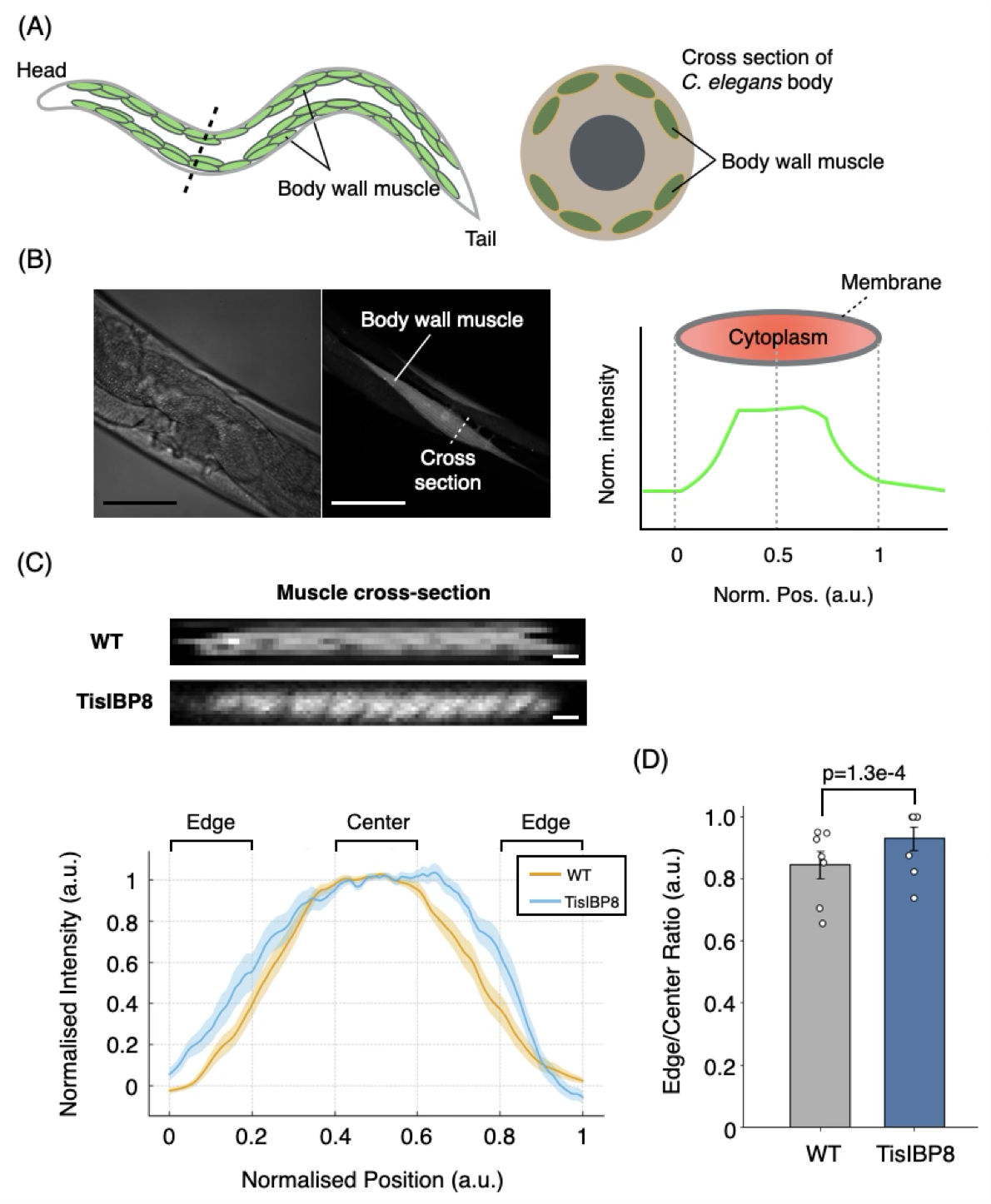


**Figure S5. Membrane-associated localization of TisIBP8 in body wall muscles of *C. elegans*.** (A) Schematic illustration of body wall muscles and a cross-section of the *C. elegans* body, highlighting the muscle layer. (B) Representative confocal images of a body wall muscle (left) and its cross-sectional view (middle). A line scan along the cross-section (right) shows the normalized fluorescence intensity with peaks corresponding to the membrane region. Representative cross-sectional fluorescence patterns are shown for WT and TisIBP8-expressing worms (bottom). The scale bar represents 50 µm. (C) The figure above shows a z-stack image of a single body-wall muscle cell; sample thickness approximately 8 µm and z-step 0.5 µm. Average fluorescence intensity profiles along muscle cross-sections. The shaded areas represent SEMs. The scale bar represents 5 µm. (D) Quantification of the edge-to-centre fluorescence intensity ratio in WT and TisIBP8-expressing worms. The data are shown as the mean ± SEM values, with the p value calculated by Student’s t test. The numbers of biological replicates were n = 7 for WT and n = 8 for TisIBP8-expressing worms.


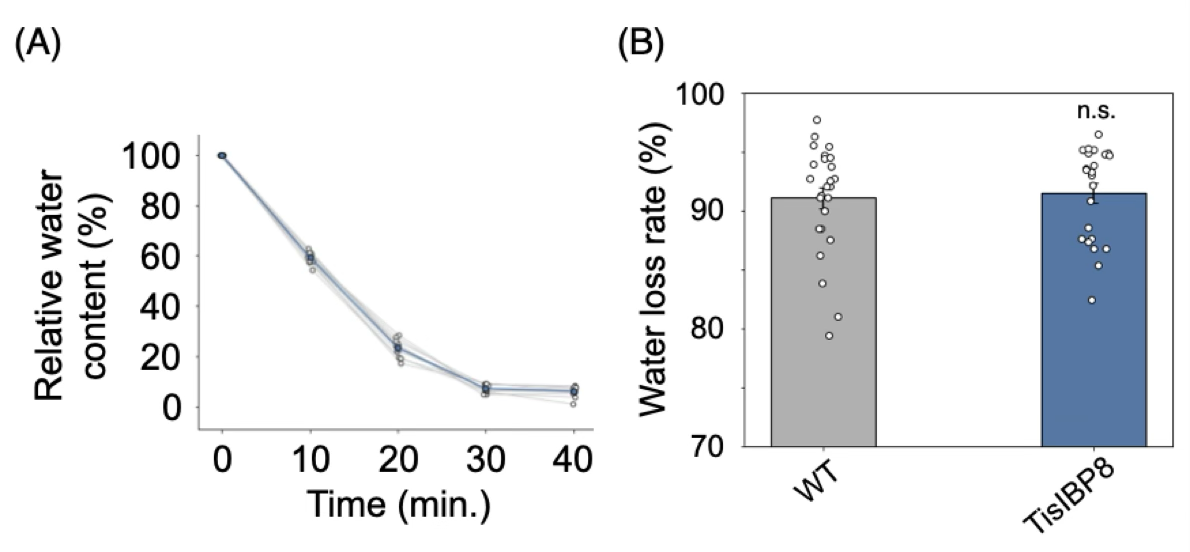


**Figure S6. Evaluation of water loss in WT and TisIBP8 worms.** (A) Water loss rate during the dehydration stress assay. Measurements were taken from 0 to 40 min. n = 10. Thick lines represent the mean values, whereas thin lines represent individual measurements. (B) Water loss rate after dehydration. This was evaluated using 50-100 worms. Data are presented as mean ± SEM. n = 25 (WT) and n = 23 (TisIBP8). Welch’s t test. n.s., not significant.

**
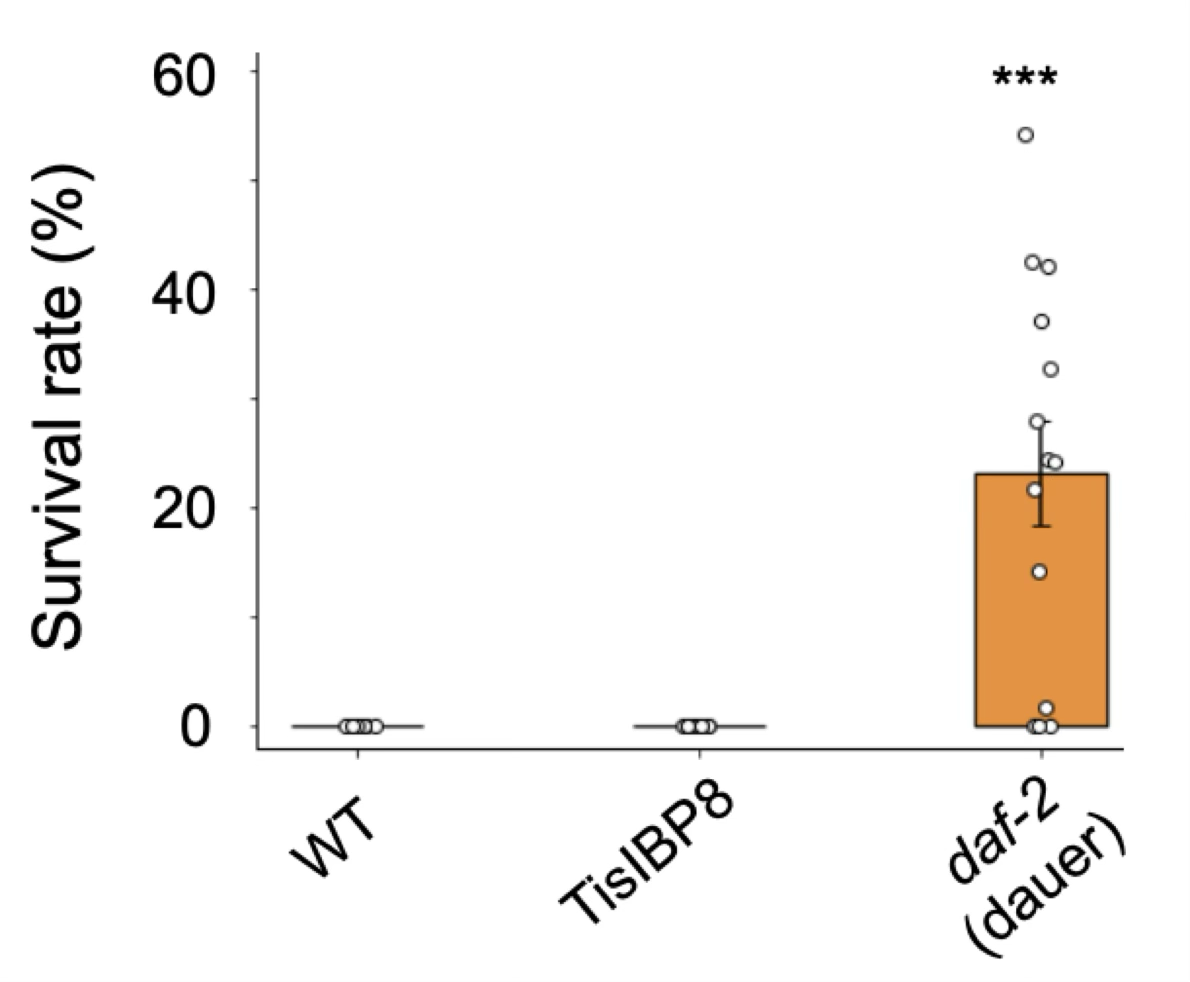
**

**Figure S7. Desiccation assay.** Survival assessment based on the conventional desiccation assay described by Erkut and colleagues [37]. Data are presented as mean ± SEM. Biological replicate numbers were n = 10 for WT and TisIBP8, and n = 14 for dauer (*daf-2*). Student’s t test. ***p< 0.001.

**Table S1. Strain List**

| Strain name | Genotype | Construct  (injection concentration) | Protein/  reference |
| --- | --- | --- | --- |
| CMS2 | *kmcEx2;*  *lin-15(n765ts)X* | *myo-3p::Venus::nfeIBP6* (50 ng/μL); *pbLH98 (lin-15(+))*  (40 ng/μL) | NfeIBP6 [42,43] |
| CMS3 | *kmcEx3;*  *lin-15(n765ts)X* | *myo-3p::Venus::nfeIBP8* (50 ng/μL); *pbLH98 (lin-15(+))* (40 ng/μL) | NfeIBP8 [42] |
| CMS4 | *kmcEx4;*  *lin-15(n765ts)X* | *myo-3p::Venus::AnpIBP* (40 ng/μL); *pbLH98 (lin-15(+))* (40 ng/μL) | AnpIBP [44] |
| CMS5 | *kmcEx5;*  *lin-15(n765ts)X* | *myo-3p::Venus::TisIBP8* (30ng/μL); *pbLH98 (lin-15(+))* (30ng/μL) | TisIBP8 [45,46] |
| CMS13 | *kmcEx13;*  *lin-15(n765ts)X* | *myo-3p::Venus::AnpIBP* (30 ng/μL); *myo-3p::NLS::wrmScarlet* (20 ng/μL); *pbLH98 (lin-15(+))* (30 ng/μL) | AnpIBP [44] |
| CMS14 | *kmcEx14;*  *lin-15(n765ts)X* | *myo-3p::NLS::wrmScarlet* (20 ng/μL); *pbLH98 (lin-15(+))* (30 ng/μL) | − |
| CMS15 | *kmcEx15;*  *lin-15(n765ts)X* | *myo-3p::Venus::AnpIBP T156Y* (30ng/μL); *pbLH98 (lin-15(+))* (30ng/μL) | AnpIBP T156Y[8,47] |
| CMS26 | *kmcEx26;*  *lin-15(n765ts)X* | *myo-3p::Venus::TisIBP8* (30ng/μL); *myo-3p::NLS::wrmScarlet* (20 ng/μL); *pbLH98 (lin-15(+))* (30ng/μL) | TisIBP8 [45,46] |
| CMS69 | *kmcEx69* | *myo-3p::wrmScarle* (60 ng/μL) | − |
| CMS73 | *kmcEx73* | *myo-3p::wrmScarlet(ΔSTOP)::TisIBP8* (150 ng/μL) | TisIBP8 [45,46] |
| CMS81 | *kmcEx81* | *myo-3p::wrmScarlet(ΔSTOP)::TisIBP8* (10 ng/μL) | TisIBP8 [45,46] |
| CMS93 | *pSAE131; Myo3p::Tis* | *myo-3p::myr-GFP* (10ng/uL)*; myo-3p::wrmScarlet(ΔSTOP)::TisIBP8* (50 ng/μL) | TisIBP8 [45,46] |
| CMS95 | *pSAE131; Myo3pwrmScarlet* | *myo-3p::myr-GFP* (10ng/uL)*; myo-3p::wrmScarlet* (50ng/uL) | − |

**Table S2. Individual survival data for WT and TisIBP8-expressing worms after 30 min of dehydration**

| No. | WT  10min | TisIBP8 10min | | WT  20min | | TisIBP8  20min | WT  30min | TisIBP8  30min | WT  40min | TisIBP8  40min |
| --- | --- | --- | --- | --- | --- | --- | --- | --- | --- | --- |
| 1 | 81.8 | 73.5 | 66.7 | | 83.3 | | 20.7 | 56.1 | 0.0 | 0.0 |
| 2 | 87.5 | 92.0 | 63.6 | | 73.9 | | 55.0 | 75.0 | 0.0 | 0.0 |
| 3 | 66.7 | 68.8 | 54.3 | | 60.0 | | 23.5 | 14.3 | 0.0 | 0.0 |
| 4 | 81.3 | 70.5 | 73.2 | | 79.7 | | 25.0 | 27.3 | 0.0 | 0.0 |
| 5 | 77.9 | 86.4 | 72.1 | | 76.9 | | 53.7 | 47.6 | 0.0 | 0.0 |
| 6 | 72.1 | 82.7 | 73.5 | | 76.3 | | 31.0 | 21.6 | 0.0 | 0.0 |
| 7 | 78.8 | 77.9 | 77.8 | | 76.3 | | 27.3 | 29.2 | 0.0 | 0.0 |
| 8 | 67.5 | 76.9 | 58.8 | | 64.0 | | 34.5 | 45.5 | - | - |
| 9 | 68.8 | 73.6 | 64.4 | | 69.6 | | 37.3 | 61.4 | - | - |
| 10 | 70.1 | 75.0 | 65.6 | | 70.4 | | 40.7 | 51.8 | - | - |
| 11 | 72.4 | 71.8 | 80.0 | | 84.0 | | 29.2 | 43.5 | - | - |
| 12 | 66.7 | 73.1 | 65.6 | | 71.4 | | 25.9 | 47.8 | - | - |
| 13 | 85.0 | 85.2 | 65.1 | | 81.8 | | 42.1 | 66.7 | - | - |
| 14 | 81.5 | 82.4 | 58.6 | | 80.9 | | 28.9 | 61.5 | - | - |
| 15 | 72.1 | 77.3 | 81.0 | | 76.3 | | 26.1 | 57.3 | - | - |
| 16 | 73.8 | 77.0 | 73.6 | | 71.4 | | 61.5 | 65.7 | - | - |
| 17 | 72.5 | 81.1 | 78.2 | | 71.4 | | 48.8 | 62.5 | - | - |
| 18 | 80.0 | 82.0 | 57.6 | | 70.3 | | 43.5 | 58.8 | - | - |
| 19 | 72.1 | 80.0 | 66.7 | | 71.7 | | 48.3 | 57.9 | - | - |
| 20 | 73.3 | 80.6 | 64.6 | | 67.4 | | 57.1 | 58.3 | - | - |
| 21 | - | - | - | | - | | 42.3 | 50.0 | - | - |
| 22 | - | - | - | | - | | 48.5 | 57.6 | - | - |
| 23 | - | - | - | | - | | 60.0 | 36.7 | - | - |
| 24 | - | - | - | | - | | 30.6 | 44.4 | - | - |
| 25 | - | - | - | | - | | 57.5 | 45.2 | - | - |
| 26 | - | - | - | | - | | 53.3 | 47.1 | - | - |
| 27 | - | - | - | | - | | 43.3 | 33.3 | - | - |
| 28 | - | - | - | | - | | 38.1 | 83.9 | - | - |
| 29 | - | - | - | | - | | 49.0 | 46.7 | - | - |
| 30 | - | - | - | | - | | 41.2 | 44.7 | - | - |
| 31 | - | - | - | | - | | - | 52.0 | - | - |

**Table S3. Individual nuclear retention data from the cell imaging analysis**

| No. | WT | | TisIBP8 | |
| --- | --- | --- | --- | --- |
|  | After 30 min.  (%) | After 60 min.  (%) | After 30 min.  (%) | After 60 min.  (%) |
| 1 | 66.0 | 26.0 | 91.3 | 28.3 |
| 2 | 98.8 | 28.6 | 93.1 | 16.1 |
| 3 | 53.7 | 17.6 | 95.5 | 18.0 |
| 4 | 76.1 | 28.3 | 93.2 | 20.4 |
| 5 | 100.0 | 51.5 | 88.3 | 21.3 |
| 6 | 92.4 | 41.9 | 100.0 | 54.4 |
| 7 | 100.0 | 22.1 | 100.0 | 19.8 |
| 8 | 82.4 | 25.5 | 95.5 | 25.0 |
| 9 | 68.1 | 34.5 | 100.0 | 33.8 |
| 10 | 72.8 | 32.6 | 100.0 | 68.0 |
| 11 | 65.5 | 9.4 | 83.5 | 28.2 |
| 12 | 57.4 | 36.1 | 96.8 | 36.6 |
| 13 | 91.3 | 29.3 | 93.2 | 39.8 |
| 14 | 88.8 | 28.6 | 95.8 | 28.4 |
| 15 | 58.8 | 28.8 | 100.0 | 37.2 |
| 16 | 51.0 | 29.4 | 69.5 | 31.7 |
| 17 | 78.2 | - | 61.5 | - |
| 18 | 76.5 | - | 82.7 | - |
| 19 | 100.0 | - | 72.8 | - |
| 20 | 100.0 | - | 83.1 | - |
| 21 | 86.4 | - | 97.1 | - |
| 22 | 98.7 | - | 84.2 | - |
| 23 | 79.5 | - | 89.0 | - |
| 24 | 81.7 | - | 100.0 | - |
| 25 | 53.0 | - | - | - |
| 26 | 58.0 | - | - | - |
| 27 | 58.0 | - | - | - |
| 28 | 64.0 | - | - | - |
